# The Basel ultrasonography protocol for assessing hepatosplenic pathologies in Asian schistosomiasis: report of a WHO expert meeting

**DOI:** 10.1186/s40249-025-01349-x

**Published:** 2025-08-08

**Authors:** Joachim Richter, Andreas Neumayr, Amadou Garba-Djirmay, Hiroshi Ohmae, Ralph Aniceto, Xiao-Nong Zhou, Jing Xu, Zhaoyu Guo, An Ning, Edward Mberu Kamau, Francesca Tamarozzi, Hannah Wei Wu, Charles King, Birgitte Jyding Vennervald, Goylette F. Chami, Jürg Utzinger, Christoph Hatz

**Affiliations:** 1 Swiss Tropical and Public Health Institute Basel, Basel, Switzerland; 2 University of Basel, Basel, Switzerland; 3 Charité University Medicine, Global Health Center, Institute of International Health, Berlin, Germany; 4 College of Medicine and Dentistry, Division of Tropical Health and Medicine, James Cook University, Townsville, Australia; 5 World Health Organization, Geneva, Switzerland; 6 Department of Parasitology, National Institute of Infectious Diseases, Tokyo, Japan; 7 Department of Tropical Medicine and Parasitology, Dokkyo Medical University, Tochigi, Japan; 8 Department of Immunology, Research Institute of Tropical Medicine, Manila, Philippines; 9 National Institute of Parasitic Diseases, Chinese Center for Disease Control and Prevention, Chinese Center for Tropical Diseases Research, Shanghai, China; 10 Jiangxi Provincial Institute of Parasitic Diseases, Nanchang, Jiangxi China; 11 Department of Infectious-Tropical Diseases and Microbiology, IRCCS Sacro Cuore Don Calabria Hospital, Negrar di Valpolicella, Verona, Italy; 12 Center for International Health Research, Rhode Island Hospital, Providence, RI USA; 13 Center for Global Health and Diseases, Case Western Reserve University, Cleveland, OH USA; 14 Section for Parasitology and Aquatic Pathobiology, Faculty of Health and Medical Sciences, University of Copenhagen, Copenhagen, Denmark; 15 Big Data Institute, Nuffield Department of Population Health, University of Oxford, Oxford, UK

**Keywords:** *Schistosomajaponicum*, *Schistosomamekongi*, Hepatosplenic schistosomiasis, Ultrasonography, Point-of-care ultrasound, Liver fibrosis, Portal fibrosis, Interseptal fibrosis, Portal hypertension, Esophageal varices

## Abstract

Asian hepatointestinal schistosomiasis due to *Schistosoma* *japonicum* is prevalent in the Philippines and in Indonesia, while it is close to elimination in China. The second Asian schistosome, *S.* *mekongi*, is found in Cambodia and Laos. The main pathology caused by both species is liver fibrosis, which can cause significant morbidity and mortality, mainly due to portal hypertension leading to bleeding from esophageal varices. Ultrasonography was introduced several decades ago as a safe, fast, non-invasive, and relatively inexpensive technique for assessing chronic schistosomiasis-related hepatic pathology in the clinical and field settings. A standardized ultrasound protocol had been established by experts at a WHO-chaired meeting in Cairo, Egypt, in 1990. The peculiarities of sonomorphologic abnormalities caused by *S.* *japonicum* and *S.* *mekongi* were not sufficiently covered in the Cairo protocol and not addressed at all in the subsequent WHO chaired meeting in Niamey 1996. At a follow-up WHO-chaired meeting in Phnom Pehnh, Cambodia, in 2002, an attempt was made to develop a protocol for Asian schistosomiasis, but a protocol resulting from this meeting has never been published. Although several studies investigated the use of ultrasonography to assess *S.* *japonicum*- and *S.* *mekongi*-related sonomorphological morbidity across endemic areas the lack of a standardized protocol hampered the characterization of sonomorphologic abnormalities with regard to progression, reversibility, prognosis, and correlation to morbidity. In addition, the comparison of data from different endemic areas and populations remained difficult. Therefore, a WHO-chiared expert meeting took place in Basel, Switzerland in September 2024 with the aim to establish a standardized ultrasound protocol for reporting the pathology caused by *S.* *japonicum* and *S.* *mekongi*. The proposed protocol is described in this article.

**Graphical Abstract:**

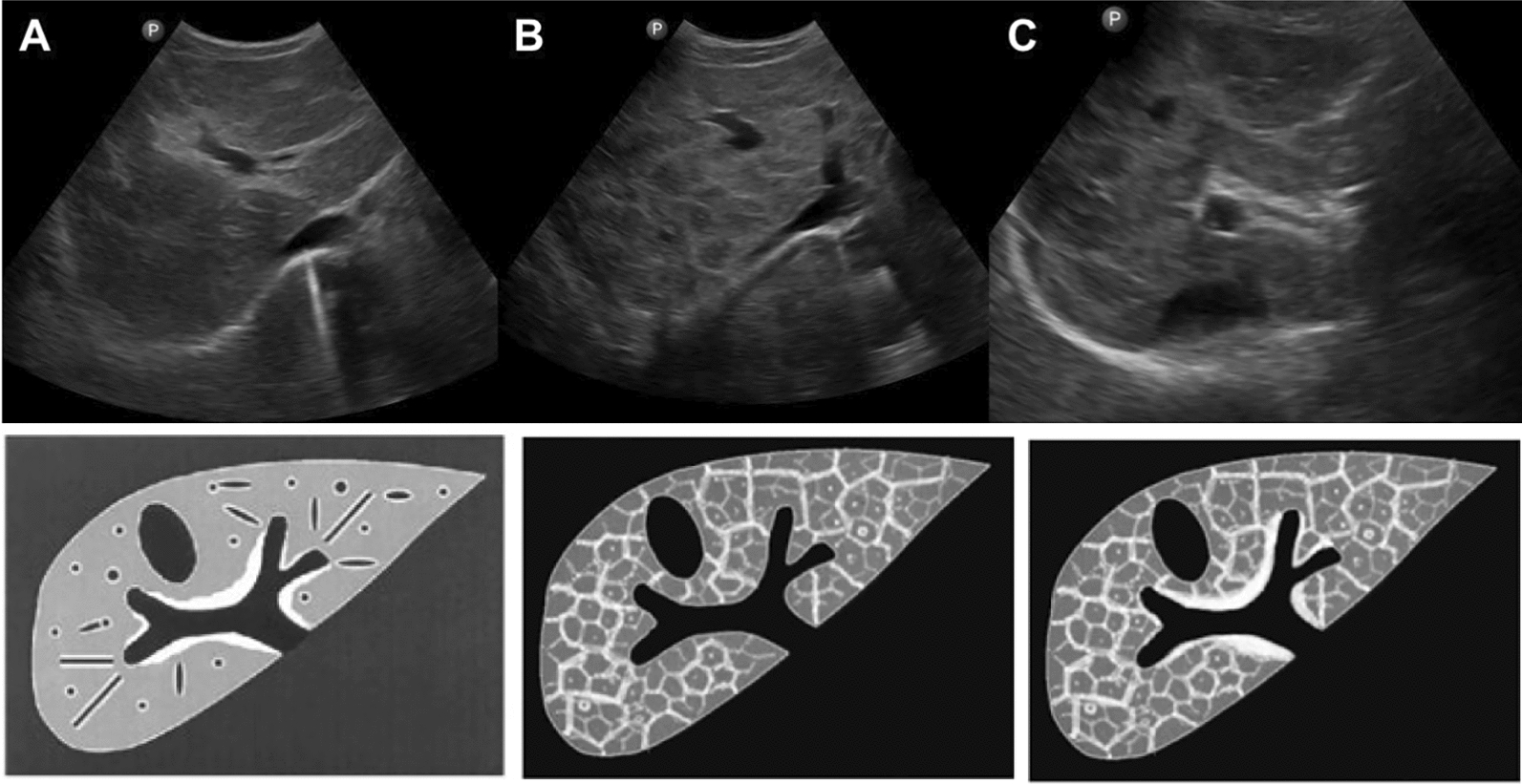

**Supplementary Information:**

The online version contains supplementary material available at 10.1186/s40249-025-01349-x.

## Background

Asian hepatointestinal schistosomiasis due to *Schistosoma japonicum* is prevalent in the Philippines and in Indonesia, while it is close to elimination in China. The second Asian schistosome, *S. mekongi*, is found in Cambodia and Laos. Apart from dramatic clinical stunting in the latter, the main pathology caused by both species is liver fibrosis, which can cause significant morbidity and mortality, mainly due to portal hypertension leading to esophageal varices bleeding.

Ultrasonography was introduced several decades ago as a safe, fast, non-invasive, and relatively inexpensive technique for assessing chronic schistosomiasis-related hepatic pathology in the clinical and field settings [1–3]. A standardized ultrasound protocol was established by experts at a WHO-chaired meeting in Cairo, Egypt, in 1990 [4]. This protocol was probably the first example of a focused ultrasound (FOCUS) or point-of-care ultrasound (POCUS) protocol. The peculiarities of sonomorphologic abnormalities caused by *S. japonicum* and *S. mekongi* were only considered to a limited extent in the Cairo protocol [4–6]. When the Cairo protocol was found to be insufficient for classifying periportal fibrosis in the 1990 s, it was revised in a WHO-chaired meeting in Niamey, Niger, in 1996. The protocol focused on *S. haematobium* and *S. mansoni*, but neither *S. japonicum* nor *S. mekongi* were included [7]. At a follow-up WHO-chaired Regional Network on Asian Schistosomiasis-meeting in Phnom Pehnh, Cambodia, in 2002, an attempt was made to develop a standardized protocol for Asian schistosomiasis, but a report and a protocol resulting from this meeting were never published. Although several studies investigated the use of ultrasonography to assess *S. japonicum-* and, to a lesser extent, *S. mekongi-*related sonomorphological morbidity across endemic areas [8–30]. The lack of a standardized protocol hampered the characterization of sonomorphologic abnormalities with regard to progression, reversibility, prognosis, and correlation to morbidity. In addition, the comparison of data from different endemic areas and populations remained difficult.

## Ultrasonography in chronic hepatosplenic schistosomiasis

Similar to chronic *S. mansoni* schistosomiasis, liver fibrosis is the most important pathology in Asian schistosomiasis. However, significant differences in fibrosis patterns are observed in Asian schistosomiasis compared to *S. mansoni* schistosomiasis. In *S. mansoni*, and possibly also in *S. mekongi* schistosomiasis, liver fibrosis almost exclusively presents as portal fibrosis (PF; syn*.* “Symmers’ fibrosis” or “clay pipe stem fibrosis”) [31, 32]. For *S. japonicum,* interseptal liver fibrosis (ISF; formerly also described as “parenchymal fibrosis”, “network fibrosis”, “turtle/tortoise back fibrosis”, “fish scale fibrosis”, “coarse reticular fibrosis”) is observed in addition to morbidity of the other schistosomal species [33–35]. This latter type of fibrosis corresponds histologically to the septa between hepatic lobules [33, 35] and is not related to the portal tract. Thus, it is different from PF. While the association between PF and portal hypertension and gastrointestinal bleeding is well established, there is no such clear association with ISF [15, 65].

**Fig. 1:**
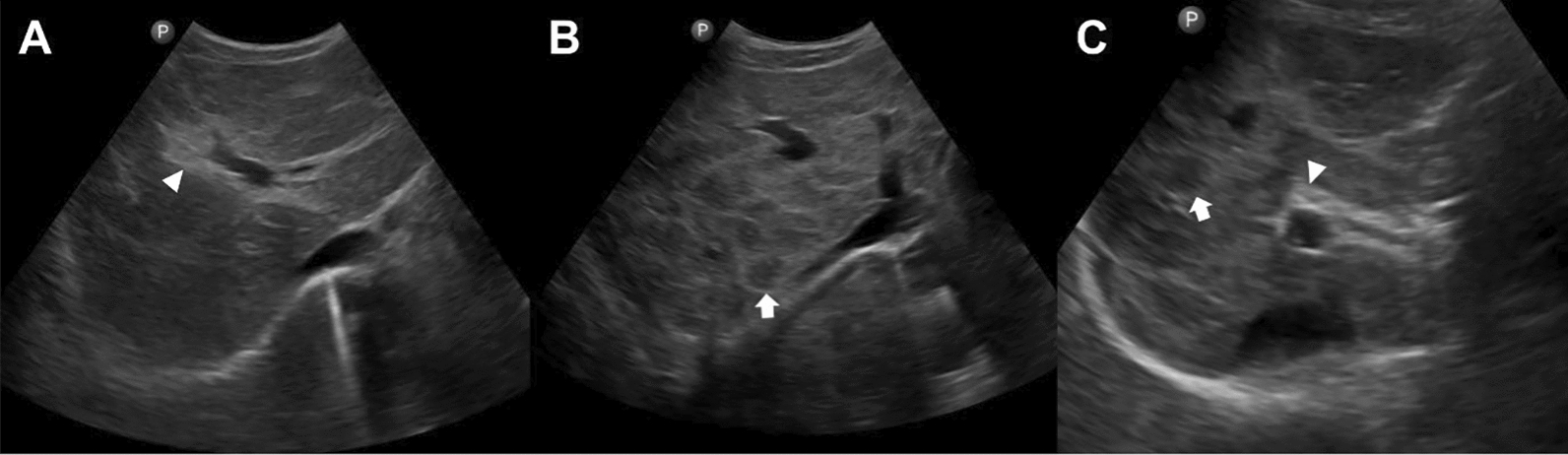
Examples of liver fibrosis patterns observed in *S. japonicum* schistosomiasis. **A** Portal fibrosis (PF; for PF other terms have been used in the literature such as “periportal fibrosis”, “clay pipe stem fibrosis”, “Symmers’ fibrosis”); ► indicates echogenic thickening of the wall of the portal vein. **B** Interseptal fibrosis (ISF; for ISF other terms have been used in the literature such as “parenchymal fibrosis”, “network fibrosis”, “turtle/tortoise back fibrosis”, “fish scale fibrosis”, “coarse reticular fibrosis”); ➨ indicates one of the polygonal "meshes" of interseptal fibrosis. **C** Concomitantly present portal and interseptal fibrosis (PF + ISF); ► indicates echogenic thickening of the wall of the portal vein; ➨ indicates one of the polygonal “network meshes” of interseptal fibrosis

PF and ISF are both well described. They may be present alone or in combination (Fig. 1). However, most studies have not separated and individually assessed the two patterns, and many authors assumed that both fibrosis patterns always coexist in advanced disease [4, 8, 9, 15, 36–41]. In addition, researchers studying Asian schistosomiasis have used either protocols which had been established for *S. mansoni* schistosomiasis, or they developed their own protocols and classifications when examining patients with Asian schistosomiasis (Table 1), which makes comparison particularly difficult. First, because ISF is not present in protocols designed for *S. mansoni* schistosomiasis, and second, because the grading of fibrosis used in different classifications do not necessarily correspond to each other.

**Table 1:** Summary of the classifications used by different authors for the grading of Asian schistosomiasis-related sonomorphologic abnormalities

Protocol/classification	Description of the grading	Type of liver fibrosis covered*	Type of liver fibrosis separated
Nayakama 1982, 1983 [37, 38]	I: Mild liver fibrosis II: Moderate fibrosis (irregular surface) III: Cirrhosis (severe fibrosis)	Non-specific fibrosis/cirrhosis	No
Uto and Nagata 1984 [38]	I: Fishscale network II: Mottled III: Sieve IV: Mixed	ISF	NA
Hannover-Managil [39]	I: Mild thickening of the wall of portal stem II: Moderate thickening + patchy fibrosis III: Patchy fibrosis extending to capsule	PF	NA
Cairo Working Group 1990 [4–7]	I: Focal echodense areas scattered within the liver parenchyma with absence of definite borders II: Stronger light bands forming a “fish scale” pattern. A few echodense areas > 2 cm III: Echodense bands forming a contiguous network. Multiple focal echodense areas > 2 cm in diameter. Masses with central fibrosis	ISF/PF	No
WHO Niamey Working Group 1996 (published in 2000) [8]	A: Normal B: “Starry sky” (diffuse echogenic foci) C: Highly echogenic “ring echoes”, which correspond to the “pipe stems” seen in a scan perpendicular to the one where rings are seen D: Highly echogenic “ruff” around portal bifurcation and main stem E: Highly echogenic “patches” extending expanding from the main portal vein and branches into the parenchyma F: Highly echogenic “bands” and “streaks”, extending from the main portal vein and its bifurcation to the liver surface, where they retract the organ surface Dc, Ec: combined patterns (D + C; E + C)	PF	NA
Disease Control Department of Chinese Ministry of Health 2000 [40], Ministry of Health, China 2006 [41]	I: Focal echodense areas II: Fishscale + mild thickening of portal walls III: Contiguous echogenic network and central echodense masses, significant thickening of portal walls and narrowing of the lumen of portal branches	ISF/PF	No
Ohmae 2003 [21]	0: Normal pattern 1: Linear pattern; some linear echogenic bands. Mild echogenic thickening of portal vein wall 4–< 6 mm 2: Tubular pattern; echogenic tubules and portal vein wall thickening ≥ 6 mm 3: Network pattern; more than 3 circles are surrounded by echogenic bands forming a fishscale/network/turtle back pattern + portal fibrosis 3a: Thin echogenic bands 3b: Thick echogenic bands	PF (1 and 2)/ISF (3)	Yes

^*^ISF interseptal fibrosis; *NA* not applicable (because only one type of fibrosis considered); *PF* portal fibrosis

The grades used in the different classifications do not not necessarily correspond to each other

## Update of the current ultrasound protocols

**Fig. 2:**
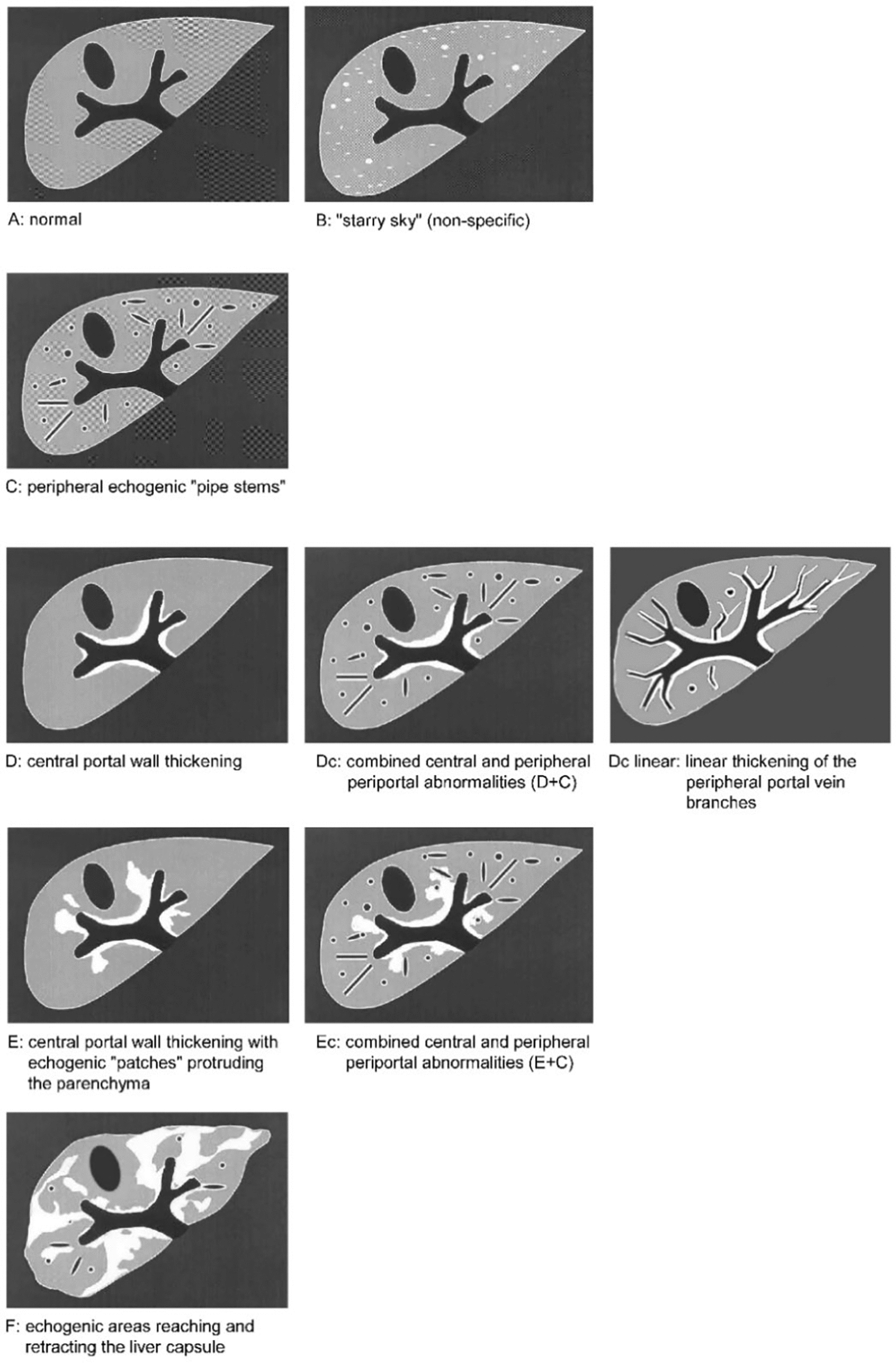
Grades of portal fibrosis (PF) in *S. japonicum*/*S. mekongi* infection. Updated pictorial/image pattern (IP) based ultrasonographic grading classification of schistosomiasis-related liver pathologies agreed upon at the WHO-chaired expert meeting held in Basel, Switzerland, in September 2024. Discrete A–F grades in this figure are identical to those agreed on at the WHO meeting in Niamey [6], for *S. mansoni* after omitting gallbladder wall thickening

**Fig. 3:**
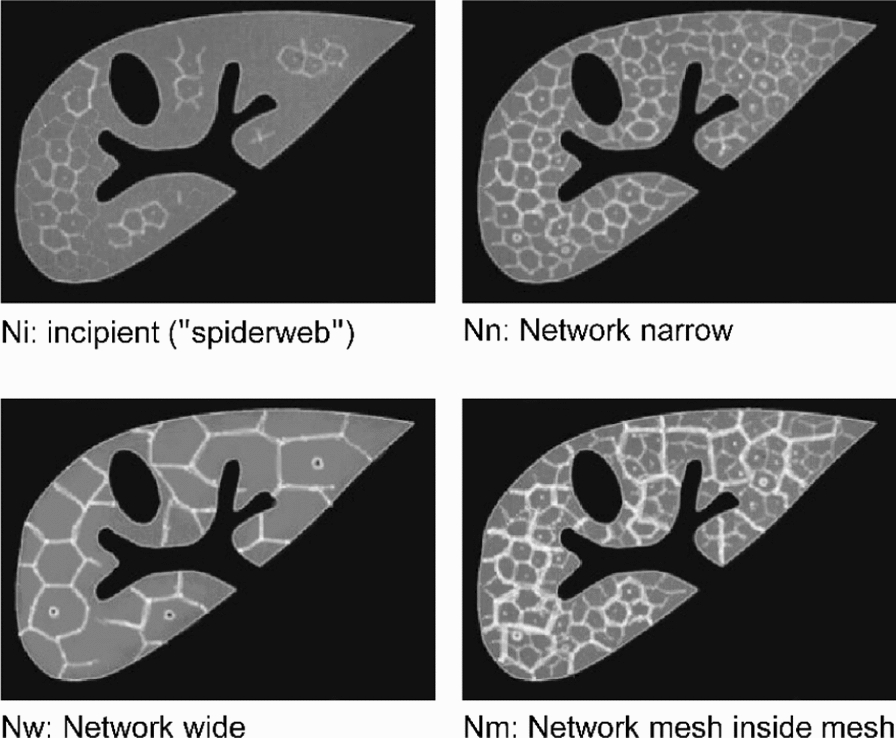
Variations of interseptal fibrosis (ISF) in *S. japonicum* infection. Updated pictorial/image pattern (IP)-based ultrasonographic variants of ISF: Ni, incipient network (“spiderweb”); so called “turtle back” network with either narrow (Nn), wide (Nw) or, "mesh-inside-mesh" (Nm) network pattern

**Fig. 4:**
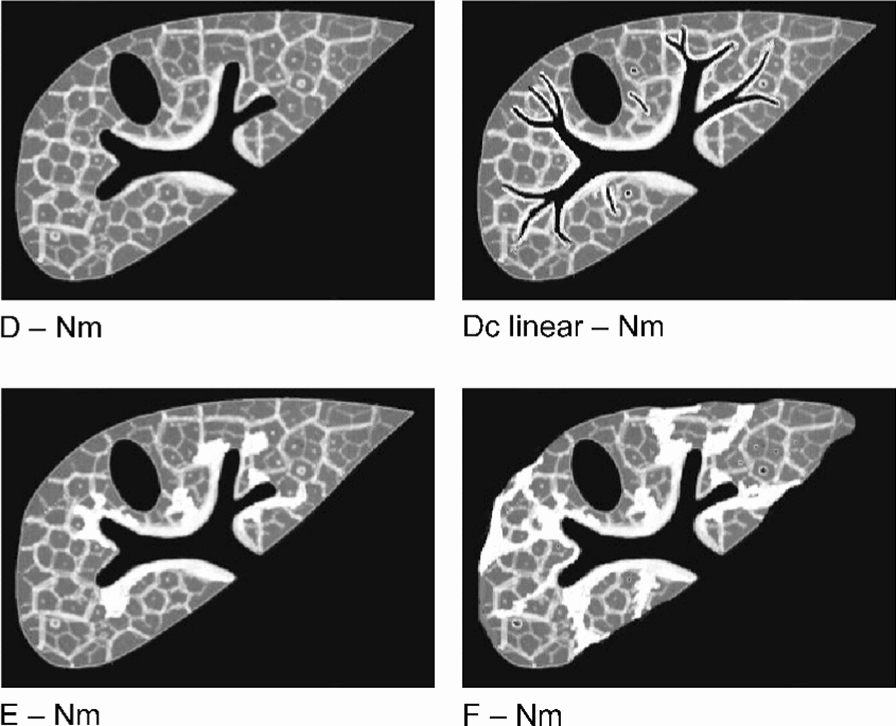
Examples of concomitant portal and interseptal fibrosis (PF + ISF) in *S. japonicum* infection. Image patterns (IP) illustrating the concomitant presence of PF and ISF. The illustrative combination depicted here are the PF grades D, Dc linear and E + the ISF variant Nm. Any stage of portal fibrosis may or may not be accompanied by any variations of interseptal fibrosis

With this document, the authors take up the sonomorphologic characteristics of Asian schistosomiasis, which were already mentioned in the first WHO ultrasound protocol formulated in Cairo in 1991 [4–6], discussed at the conference in Phnom Penh in 2002 (unpublished conference proceedings) and only recently taken up again at a WHO-chaired expert meeting in Basel, Switzerland, in September 2024. Here, we present the agreement reached at the meeting in Basel, focusing on ultrasound image patterns (IP) of Asian schistosomiasis-related liver pathologies and the clinical translation of ultrasonography findings, including “danger signs”. Figures 2, 3, 4 show the updated pictorial ultrasonographic classification agreed upon at the Basel meeting.

The two left rows of image patterns (grades A–F) in Fig. 2 are identical to those agreed on at the WHO meeting in Niamey [6] for *S. mansoni,* with the modification that no gallbladder wall thickening is seen in Asian schistosomiasis. The newly added IP “Dc linear” depicts a special fibrosis pattern, not seen in *S. mansoni* but frequently in *S. japonicum* schistosomiasis [21, 42]. This pattern is characterized by linear thickening of the peripheral portal vein branches extending from the central to the peripheral part of the liver. The inclusion of this IP is intended to supplement and refine the classification of PF in Asian schistosomiasis.

Figure 3 depicts IPs corresponding to variants of interseptal fibrosis (ISF) commonly seen in *S. japonicum* infection. Note that since the natural history of ISF development is currently not fully elucidated, we intentionally used the term “variants” instead of “grades”. Any grade of PF may or may not be accompanied by any variation of ISF.

Figure 4 shows examples of concomitant PF and ISF. For reasons of clarity, we have deliberately refrained from presenting all possible combinations.

Currently, there is very limited evidence that isolated ISF is correlated with liver disease and its resulting complications [20, 45, 2] (compiled by An Ning and Joachim Richter) comparatively lists the corresponding grades of the classification currently used in China [40, 41] and examples of how these would be described according to the grading proposed by the Basel classification.

**Table 2:** Grading according to the current Chinese ultrasonography classification and the putatively corresponding grades according to the proposed Basel ultrasonography classification

Current Chinese ultrasonography classification [40, 41]		Corresponding PF grades and ISF variants according to the proposed Basel ultrasonography classification			
Grade	Description of ultrasonographic abnormalities^1^ [40, 41]	ISF^1^			PF^1^
I	Focal echodense areas	nq	+		B
II	Fishscale^2^ + mild thickening of portal walls	Nn Nw		+ ^3^	C
III	Contiguous echogenic network and central echodense masses, significant thickening of portal walls and narrowing of the lumen of portal branches	Nn Nw Nm		+ ^3,4^	D, Dc, Dc linear E Ec F

^1^*ISF* interseptal fibrosis, *PF* portal fibrosis

^2^fishscale = another denomination for an aspect of ISF

^3^note that the current Chinese ultrasonography classification does not differentiate between PF and ISF and that all three grades describe the concomitant presence of both entities (as e.g. in Fig. 4). Whereas the Basel classification can describe all grades of the Chinese CDC classification, this is, vice versa not the case

^4^note that any combination of the listed ISF variants with any of the listed PF grades would comply

## Danger signs

It is crucial to appreciate the occurrence of severe morbidity in a patient who may be at high risk of a dangerous course of the disease, including death. Therefore, ultrasonographic “danger signs” were newly defined. They correlate with the risk of complications occurring and require timely referral of patients. These “danger signs” [6, 43–46] include:

- advanced portal fibrosis, i.e. IP Dc, E, Ec, F
- portal vein dilatation [i.e. a portal vein quotient (PVQ) > 7.5 mm/m (portal vein diameter in mm/patient’s height in m)]
- presence of porto-systemic collaterals
- presence of ascites

The danger signs have been evaluated mostly for schistosomiasis mansoni and liver cirrhosis. There is a need of further investigations for Asian schistosomiasis.

## Organometry

Measurements were reduced to those deemed essential, mainly portal vein diameter at the entry point into the liver. Other measurements are optional. Reliable measurements must be adjusted for body height (i.e. PVQ) [44, 47].

## Portal vein quotient (PVQ)

For a rapid estimation of the risk of bleeding from esophageal varices, the portal vein quotient (PVQ; Table 3) is calculated by dividing the diameter of the portal vein (in mm) by the body height of the subject (in m); this has shown to predict the risk of bleeding in field studies on *S. mansoni* [46].

**Table 3:** Portal vein quotient (PVQ)

Portal vein quotient (PVQ) (mm/m)	Interpretation	Score value	Bleeding risk (% of cases)
≤ 7.5	Normal	0	0
˃ 7.5–10	Dilated	1	20–50
˃ 10	Severely dilated	2	≥ 70

## Schistosoma risk score (SRS)

For *S. mansoni*, a risk score compiled from the IP and the PVQ has been prospectively tested regarding its predictive value to judge the risk of gastro-intestinal bleeding [46]. There is a need of further studies to assess if this score may also be used for *S. japonicum* and *S. mekongi* morbidity [15, 45, 65]. The SRS value is calculated by summing up the IP score value (Table 4) and the PVQ score value (Table 2). The SRS can vary from 0–4. The approximate risk of re-bleeding in a Brazilian cohort within 1 to 4 years was 0% for scores 0 and 1, 10% for score 2, 90% for score 3 and 100% for score 4 [46].

**Table 4:** Schistosomiasis risk score (SRS)

Image pattern (IP)	Score value		Portal vein quotient (PVQ) (mm/m)	Score value		SRS
A, B, C, D	0		≤ 7.5	0		0–4
E	1	+	˃ 7.5–10	1	=	
F	2		˃ 10	2		

## Optional measurements/assessments

- Spleen: splenomegaly is a useful parameter for assessing early hepatosplenic schistosomiasis (reactive splenomegaly). Any measurement must be adjusted to body height, which is therefore an important data to record [47–49].
- Gallbladder: typical echogenic wall thickening (≥ 4 mm in a patient fasting more than 8 h) with a smooth internal surface and external protrusions can be observed, frequently in continuation with periportal thickening. However, gallbladder involvement is less frequent in Asian schistosomiasis as compared to schistosomiasis mansoni [42, 50]. In schistosomal cholecystopathy, the gallbladder is not tender at ultrasound-guided palpation and usually does not contain calculi. There are occasional case reports describing gallbladder polyps [51]. In co-endemic areas involvement of the biliary system by co-morbidities (flukes, gallstones, bacterial cholecystitis, malignancy) should be considered in the differential diagnosis [52–55].

## Differential diagnosis/co-morbidities

**Fig. 5:**
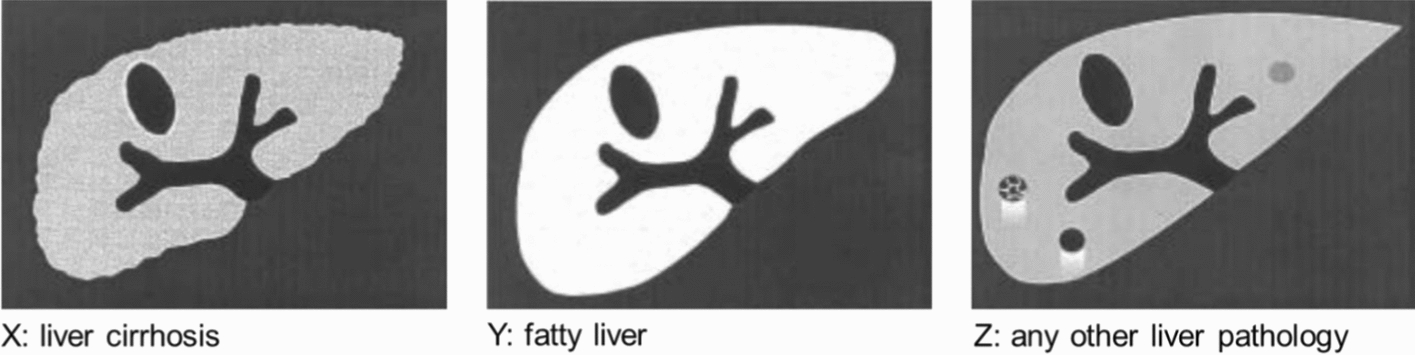
Image patterns (IP X, Y, Z) of differential diagnoses

- When biliary morbidity is present, co-infections with liver flukes (fascioliasis, opisthorchiasis/clonorchiasis) should be considered [30, 56–62]. This applies particularly to schistosomiasis mekongi in Laos [30, 62]. Differential diagnosis between portal fibrosis of peripheral portal branches seen in schistosomiasis from periductal fibrosis in liver fluke infections may sometimes be difficult. Color Doppler scanning revealing intraluminal blood flow inside the portal branches with thickened walls as observed in schistosomiasis may be helpful in differentiating these from peripheral bile duct with thickened walls in opisthorchiasis [61].
- In addition, the contribution of noxious agents (e.g. alcohol or toxins) and metabolic disorders which may cause fatty liver (IP Y) or cirrhosis (IP X) (Fig. 5) should be evaluated [63].
- Co-morbidity due to chronic viral hepatitis (HBV, HCV) is frequent and may cause cirrhosis and worsen schistosomal fibrosis (IP X) [42, 64].

## Duplex-/Doppler ultrasonography

Many new portable US devices allow performing Doppler ultrasonography [15, 61, 65, 66]. Color-Doppler US is particularly useful for identifying collateral vessels and for differentiation of portal branches from bile ducts [61]. When interpreting Continuous-Wave-Doppler (CW-Doppler) results, it must be taken into account that in PF, contrary to liver cirrhosis, portal flow velocity is not reduced but increased because of hyperafflux due to hypersplenism resulting from hyperreactive splenomegaly, and flow is not inverted [66]. This implies the risk of underestimating portal hypertension in schistosomiasis.

Of note is also, that portal flow is increased postprandially. Doppler Ultrasound measurements are found abnormal in PF but not alterated in ISF [15].

## Elastography of liver and spleen

Elastography of the liver is useful for identifying schistosomal liver fibrosis mansoni [67]. When reporting elastography results, the method used needs to be quoted, e.g. “Fibroscan” or Acoustic Radiation Force Impulse Imaging (ARFI). Unfortunately, there is no distinction of the corresponding type of liver fibrosis (ISF or PF). Further comparative studies are warranted to better characterize which type of fibrosis is linked to which liver and/or spleen stiffness. Since the liver parenchyma outside the portal tracts is not affected in PF, liver stiffness may be less increased than in liver cirrhosis. On the other hand, elastography of the spleen could especially add to the diagnosis of portal hypertension in schistosomiasis [67–69].

## Summary

This proposed ultrasound evaluation and grading protocol of schistosomiasis-related liver pathologies in Asian schistosomiasis updates former protocols [4–7, 40, 41].

## Supplementary Information

Supplementary Material 1.

## Data Availability

The online version contains supplementary material available at 10.1186/s40249-025-01292-x.
