# Supplementary material for: The Basel ultrasonography protocol for assessing hepatosplenic pathologies in Asian schistosomiasis: report of a WHO expert meeting
Source: Infect Dis Poverty. 2025 Aug 8;14:83. doi: 10.1186/s40249-025-01349-x (PMC12333150; doi:10.1186/s40249-025-01349-x)
Supplement: Supplementary file 1 — Supplementary Material 1. [file 40249_2025_1349_MOESM1_ESM.pdf]

Case report form: *S. japonicum/S. mekongi*-associated ultrasonography findings – standard examination

Pat. No. Sex: ☐ female ☐ male Date of examination: ___ /___ /_______

Surname: Given name:

Age (years): _ _ Height: ____cm Fasting since: _ _ hours

HBsAg status: ☐ positive ☐ negative ☐ not known Liver fluke co-infection: ☐ yes ☐ no ☐ not known

History: vomited blood? ☐ yes ☐ no ☐ not known observed black stools? ☐ yes ☐ no ☐ not known

Any other symptom? ☐ yes ☐ no If so; which?

LIVER

Measures (Portal vein diameter mandatory; PSL and MCL optional)

Portal vein diameter: _____ mm Liver span in PSL_____mm in MCL______mm

Image Pattern (IP): ☐ **A** ☐ **B** ☐ **C** ☐ **D** ☐ **E** ☐ **F**
☐ **Dc*linear** ☐ **Ec***
☐ **Dc*** (*subgrade both D or E and C)

Subgrading peripheral periportal fibrosis **B** ☐ ☐ ☐ **C** ☐ ☐ ☐

Subgrading central periportal fibrosis **D** ☐ ☐ ☐ **E** ☐ ☐ ☐ **F** ☐ ☐ ☐

ISF variant of interseptal (network / fishscale / turtle back) fibrosis ☐ **nq** ☐ **Ni** ☐ **Nn** ☐ **Nw** ☐ **Nm**
nq = questionable incipient lesion; Ni = incipient spider web;
Nn = narrow meshes < 2 cm ; Nw = wide meshes 2 cm and more; Prevalent mesh size unable to decide ☐
Nm = mesh inside mesh

Subgrading of network lesions weak strong
☐ ☐ ☐

☐ **X** (Cirrhosis-like) ☐ **Y** (Steatosis-like) ☐ **Z** (other pathology), specify:______________________

☐ Irregular liver surface Caudal liver edge: ☐ sharp ☐ rounded

BILIARY SYSTEM *If abnormal check for liver flukes.*

Gall bladder wall thickened >4 mm	☐ no	☐ yes
Gall bladder wall external protrusions	☐ no	☐ yes
Content sludge / stones / polyp	☐ no	☐ yes, specify:______________________
Tenderness under palpation	☐ no	☐ yes
Peripheral bile duct dilatation	☐ no	☐ yes
Common bile duct (CBD) dilatation	☐ no	☐ yes

SPLEEN *(optional).*

Length: ________mm Depth: ________mm Splenic vein diameter at splenic hilum: _____mm

PORTAL HYPERTENSION / SPECIFIC DANGER SIGNS *If any danger sign is present, refer to a clinician.*

Adanced periportal fibrosis (IP: Dc, E, Ec, F):	☐ no	☐ yes
Portal vein dilatation, PVQ >7.5 mm/m:	☐ no	☐ yes: PVQ: ___ mm/m:<br>Portal vein diameter: ____ mm<br>Height: __,__ m
Presence of porto-systemic collaterals:	☐ no	☐ yes
Presence of ascites:	☐ no	☐ yes

Observations

Explanations to the case report form

1. Liver span measurements are optional because the way as these are measured are influenced by the constitution of the person scanned. In over-weight patients the span underestimate liver size. Liver span measurements may be useful in children with incipient hepatosplenic schistosomiasis (see 1.1.)
2. Portal stem measurements adjusted to height are crucial for assessing portal hypertension. Portal vein diameter increases after food intake. Therefore, the subject should be fasting since at least four hours
3. Image pattern (IP) A means a normal liver image. IP B is an aspect where the peripheral portal walls are unusually well seen ("starry sky"). It might indicate incipient portal fibrosis but it is also frequently seen in healthy children and other conditions like stving or febrile viral infections.
4. IP's C, D, Dc, Dc linear, E, Ec and F indicate portal fibrosis.
   4.1. These may concomitantly with any IP's reflecting interseptal fibrosis.
   4.2. The possibility of sub-grading is offered in order to reduce pseudo-inter-observer variance and for refining post-therapy dynamics
5. IP's N indicate interseptal fibrosis. nq means network questionable incipient fibrosis similar as IP B for portal fibrosis; Ni stands for incipient spider web; Nn for narrow meshes <2 cm ; Nw for meshes 2 cm and more; Nm for mesh inside mesh network. Any IP for portal fibrosis may occur concmitantly with and IP for interseptal fibrosis. Subgrading is offered as for IP's for portal fibrosis. For the thickness of the strongest interseptal line: < 2mm = weak; 2 to 4 mm = intermediate; > 4mm = strong.
6. IP's X,Y,Z may be observed alone or in any combination with other IP's (except for IP A). These patients have to be judged separately. Patients with IPX combined with portal fibrosis are of particular risk of variceal bleeding and ascites.
7. Irregular liver surface is usually observed in patients with liver cirrhosis, as well as in patients with advanced portal and interseptal fibrosis.
8. Rounded caudal liver edge in the left parasternal line usually indicates fatty liver (IP Y) observed most frequently in overweight patients, diabetes and hyperlipoproteinemia as well as with alcohol abuse.
9. Biliary abnormalities in Asian schistosomiasis may sometimes accompany portal fibrosis ("schistosomal cholecystopathy"). For scanning the gallbladder the patient must be fasting and not drinking coffee (there are no data for black or green tea) since 6-8 hours, because otherwise the gallbladder may be contracted or present with pseudo-wall thickening. Typically, the gallbladder wall is echogenic, thickened, not tender at ultrasound-guided palpations and not associated with gallbladder stones. The bile ducts are normal. Schistosomal cholecystopathy is much rarer in Asian schistosomiasis in comparison to schistosomiasis mansoni. Some publications report on gallbladder polyps. Generally, biliary abnormalities point at liver fluke co-infections especially in *S. mekongi* infections.
10. Spleen measurements are optional but may, especially in malaria-free areas be useful.
    10.1. In very early infections hyperreactive splenomegaly may indicate hepatosplenic schistosomiasis before liver fibrosis is clearly identifiable. The dynamics of spleen size may also be useful for documenting the impact of therapy
    10.2. An enlarged splenic vein and reduction of variation of splenic vein

diameter are indicators of portal hypertension.

10.3. As spleen measures length and depth were preferred to volume because less subject to inter- and intra-observer variance than volume

11. Portal hypertension is reflected by a dilated portal and splenic vein, splenomegaly, porto-systemic collateral vessels and/or ascites. These patients are at high risk of complications. Portal hypertension signs and IP’s of Dc and higher constitute “danger signs” for lethal gastro-intestinal bleeding.
